# Evaluation of Immunological Parameters in Purified Protein Derivative Positive Tuberculin Workers 

**Published:** 2013-09

**Authors:** Shohreh Azimi, Majid Tebianian, Nader Mosavari, Azar Sabokbar, Farhad Jalali, Saba Arshi, Reza Arefpajouhi

**Affiliations:** 1 Department of Microbiology, Faculty of Science, Islamic Azad University, Karaj Branch, Karaj, Iran; 2 Razi Vaccine and Serum Research Institute, Karaj, Iran; 3 Department of Allergy and Clinical Immunology, Rasoul Akram Hospital, Tehran University of Medical Sciences, Tehran, Iran

**Keywords:** Cytokine, Immunological responses, Lymphocyte population, PPD positive

## Abstract

***Objective(s):*** According to the occupationally risk of infection in staff workers who have direct contact with mycobacterium species, we investigated their immunological parameters and compared with healthy purified protein derivative (PPD) negative volunteers.

***Materials and Methods*** *:* We investigated 20 PPD positive volunteers working at Tuberculin Unit of Razi Vaccine and Serum Research Institute and PPD negative healthy controls with no exposure or history of active tuberculosis. The percentages of circulating lymphocyte subpopulations were detected by flowcytometry. IL-4 and IFN-γ production levels were measured by ELISA in supernatants of PPD-stimulated peripheral blood mononuclear cells (PBMCs) culture.

***Results*** *:* Tuberculin workers showed an increase in IFN-γ level and significant decrease of CD4+ T cells percentage and CD4/CD8 ratio compared to PPD negative normal individuals. However the IL-4 production and percentage of other lymphocyte population has been unchanged.

***Discussion:*** These observations suggest that the immunological parameters of tuberculin workers with PPD positive reaction, who are occupationally exposed to mycobacterium antigens, could be changed. Future studies will be directed towards cytokine networking and regulatory lymphocytes, which will help us validate the significant data presented in this study.

## Introduction

Tuberculosis, caused by *Mycobacterium tuberculosis*, is a major health problem which has beensignificantly increased during recent years (1).

Historically, tuberculin skin test (TST) remains as the most common diagnostic method for evaluation of previous contact with mycobacteria or tuberculosis infection (2, 3).

In this test, delayed type hypersensitivity response to intradermal injection of purified protein derivates (PPD) was analyzed and Interpretated within 48-72 hr (3, 4).

The diagnostic potential of TST for *M. tuberculosis* infection is not well defined and this is considered as a poorly sensitive method. False positive results may be determined in BCG vaccinated or healthy medical workers who are exposed to environmental or non tuberculosis mycobacteria (3, 4).

Limited results on immunological parameters of healthy PPD positive workers exist. According to the occupational risk of infection in staff workers who have direct contact with mycobacterium species, we investigated their immunological parameters and compared them with healthy PPD negative volunteers.

## Materials and Methods

***Participants***

Study population consists of twenty (16 male and 4 female) healthy workers at tuberculin unit of Razi Vaccine and Serum Institute with positive reaction to PPD. All of them have potential contact with mycobacterial antigens with no history of active tuberculosis. Twenty five (18 male and 7 female) healthy subjects who had negative results of PPD skin test were selected for controls. The average age of subjects was 36.2 years.

All participants consented to take part in this study and ten milliliter of heparinized peripheral blood was collected from them.

***Peripheral blood mononuclear cells (PBMCs) isolation, culture and cytokine assay***

PBMCs were isolated by Ficoll-hypaque density gradient centrifugation. The cells were washed and finally suspended in complete RPMI-1640 medium (10% human antibody serum + 100 u penicillin-streptomycin/ml) and counted.

A total of 1×10^6^ cells/well was cultured in flat-bottomed 24 well plates in duplicate with or without PPD (10 μg/ml).

The cultured plated were incubated at 37°C in an atmosphere of 5% CO_2_. After 4 days the supernatants were recovered and stored at -70°C.

The concentration of IFN-γ and IL-4 were measured using commercial anzyme linked immunosurbent assay kit (R&D) according to manufacturer’s specifications. 

***Flowcytometry procedure***

T and B lymphocyte and natural killer (NK) cells surface markers were enumerated by two color flow cytometry. For this reason a panel of monoclonal antibodies whish was conjugated with fluorescein isothiocyanate (FITC) and phycoerythrin (PE) consisting of HLA-DR, CD14, CD19, CD3, CD4, CD8, CD22, CD16+CD56 (all from DAKO) were used. 

Peripheral blood samples were transported in sodium heparin tubes and were stained with a combination of monoclonal antibodies.

After incubation, washing and fixation, samples were quantified in a partec flowcytometer. 

Data analysis was performed using winMDI 2.9 software and percentage of each marker expression was determined based on events in lymphocyte gate.

***Statistical analysis***

Data were reported as the mean ± standard error of the mean (SEM). Comparisons between groups were usually performed using unpaired two-tailed Student’s t-test. A difference was considered to be statistically significant for a *P* value lower than 0.05 (*P*< 0.05)

## Results

***Lymphocyte analysis***

To determine the lymphocyte subpopulation, we analysis the surface expression of lymphocyte markers with flow cytometry.

According to Table 1, data showed that PPD-positive tuberculin workers have a higher percentage of CD3+/CD8+ positive T lymphocytes compared to PPD-negative controls (38.33 ± 7.61 vs. 27.63±8.11, *P*<0.05). The percentage of CD3+/CD4+ positive T cells was slightly lower in PPD-positive group (41.66±5.94 vs. 45.27±7.51, *P*>0.05). However, the CD4/CD8 ratio was declined.

There were no differences in the percentage of B lymphocyte expressing CD22 (6.33 ± 3.11 vs. 7.24 ± 3.87, *P*>0.05) and NK cells expressing CD16 and CD56 molecules (10.76±3.21 vs. 11.63±2.6, *P*>0.05) in two separated groups (Table 1).

**Figure 1:**
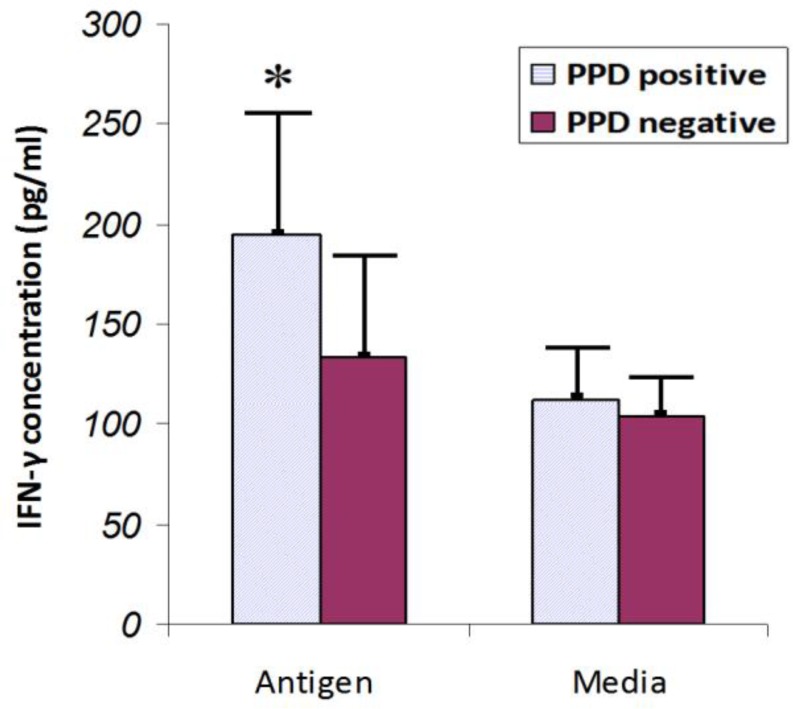
Comparison of IFN-γ levels in cultures of lymphocytes stimulated with antigen (PPD) or without antigen (Media). The cytokine level was measured 4 days after antigen stimulation. * The level of IFN-γ showed a significantly increase in the PPD positive subjects compared with negative ones (*P*< 0.05). Results are represented as the mean ± SD

**Figure 2:**
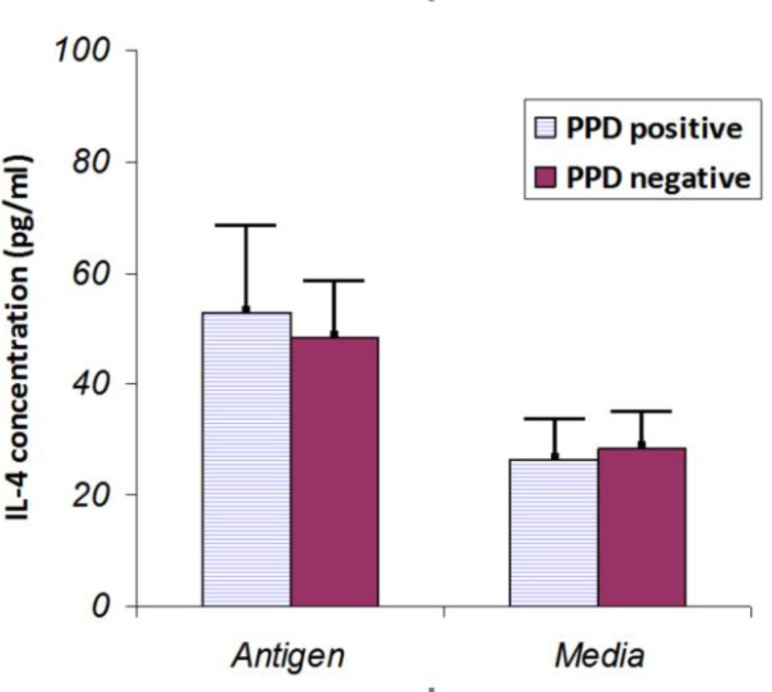
Comparison of IL-4 levels in cultures of lymphocytes stimulated with antigen (PPD) or without antigen (Media). The cytokine level was measured 4 days after antigen stimulation. The level of IL-4 showed a slightly and non-significantly increase in the PPD positive subjects compared with negative ones (*P *> 0.05). Results are represented as the mean ± SD

**Table 1:** Percentage of selected lymphocyte markers in PPD positive and negative subjects

Lymphocyte marker	PPD positive	PPD negative
CD3+/CD4+	41.66 ± 5.94	45.27 ± 7.51
CD3+/CD8+	38.33 ± 7.61	27.63 ± 8.11
CD4/CD8 ratio	1.09 ± 0.24	1.54 ± 0.36
CD22+	6.32 ± 3.11	7.24 ± 3.87
CD16+/CD56+	10.76 ± 3.21	11.63 ± 2.6

***Cytokine production by PBMCs***

PBMCs from PPD-positive tuberculin workers and controls were cultured in the presence of PPD antigen and the levels of IFN-γ and IL-4 production was assayed by ELISA.

The results showed that, IFN-γ concentration in PPD-positive subjects was significantly greater than that of PPD-negative controls (194.41 ± 53.34 vs. 133.11 ± 40.6, *P* <0.05) (Figure 1).

Furthermore, production of IL-4 was similar in PPD-positive and negative groups (52.93 ± 10.06 vs. 48.38 ± 12.2, *P* >0.05) (Figure 2).

## Discussion

Tuberculosis is endemic in Iran and many individuals are sensitized to tuberculosis or non-tuberculosis mycobacterial antigens. This is revealed by positive skin reaction to PPD antigens. 

However, to our knowledge, no report concerning the immune responses pattern of PPD positive healthy workers who have consistent exposure to mycobacterial antigens exist. They are good subjects for study of immune responses in non-tuberculosis mycobacteria (NTM).

Based on the majority of reports, mycobacteria infection could predominantly induce Th1 cells and CD8^+^ cytotoxic T cells with a Th1-like cytokine profile of elevated IFN- levels (6-8). Here, we studied the lymphocyte subpopulation and PPD-specific cytokine production in PPD positive subjects who have been worked in tuberculin production unit of Razi Vaccine and Serum Research Institute (RVSRI).

In the first step of this study, the lymphocyte subpopulation was determined by double-color flow cytometry. Based on our findings, the percentage of CD8^+^ lymphocytes were significantly greater in the PPD positive tuberculin workers compared to PPD negative control group. The CD8+ lymphocytes have an important roles for defense against intracellular pathogens, such as mycobacteria, and several studies are reported on increase of these cells in both tuberculosis infected or PPD positive subjects (8-10).

However, in this study no difference was observed in CD4+ lymphocytes. This could emphasize the role of mycobacterial antigens for over stimulating and increase of CD8+ lymphocytes and shifting of immune responses toward theses cells.

IFN-γ is a crucial cytokine for controlling of intracellular infection. This cytokine could be secreted from activated TH1 and CD8+ or NK cells (11- 13).

In agreement with previous studies (12, 13), the present results show that IFN-γ production by PBMCs were greater in the PPD-positive donors in response to PPD antigen compared to the controls. However, no signiﬁcant difference was found between PPD positive and control groups for production of IL-4, as an important cytokine that down-regulates Th1 immune responses. This is contradicting with some previous studies that reported the elevated levels of IL-4 in lymphocytes stimulated by *mycobacterial* antigens (5, 13).

## Conclusion

Overall, based on these data, we suggest an initial dominant Th1 response with elevated IFN-γ and CD8+ T cells count in PPD positive individuals who are constantly affected by mycobacterial antigens. This may be responsible for the elevated cell mediated immunity in these individuals and could interfere with some potentially pathologic criteria or diseases such as autoimmunity or allergic reaction. This could be mentioned for future studies.
